# Arts therapies for mental disorders in COVID-19 patients: a comprehensive review

**DOI:** 10.3389/fpubh.2023.1289545

**Published:** 2023-12-04

**Authors:** Xuexing Luo, Zheyu Zhang, Xiaojun Shi, Caihong He, Jue Wang, Qibiao Wu, Guanghui Huang

**Affiliations:** ^1^Faculty of Humanities and Arts, Macau University of Science and Technology, Taipa, Macau SAR, China; ^2^Department of Hepatology, The 4th People's Hospital of Qinghai Province, Xining, Qinghai, China; ^3^Operation Management Centre, Guangzhou Wanqu Cooperative Institute of Design, Guangzhou, Guangdong, China; ^4^State Key Laboratory of Quality Research in Chinese Medicines, Macau University of Science and Technology, Taipa, Macau SAR, China; ^5^Faculty of Chinese Medicine, Macau University of Science and Technology, Taipa, Macau SAR, China; ^6^Guangdong-Hong Kong-Macao Joint Laboratory for Contaminants Exposure and Health, Guangzhou, Guangdong, China

**Keywords:** mental disorders, COVID-19, Post-Acute COVID-19 Syndrome, arts therapies, psychotherapy

## Abstract

**Background and objective:**

The COVID-19 global pandemic has necessitated the urgency for innovative mental health interventions. We performed a comprehensive review of the available literature on the utility and efficacy of arts therapies in treating mental health problems, with special emphasis on their deployment during the COVID-19 pandemic, aiming to provide some evidence for the application of this therapy.

**Methods:**

The potential studies were systematically sourced from five authoritative databases: PubMed, Embase, the Cochrane Library, Web of Science, and the CNKI database. The evaluation of these studies was conducted based on stringent criteria, including validity, suitability, therapeutic potential, and consistency. Each piece of included literature was meticulously scored in accordance with these criteria, thus ensuring the inclusion of only the most robust studies in this review. The data from these Randomized Controlled Trials (RCTs) were carefully extracted using the PICO(S) framework, ensuring a comprehensive and systemic approach to data collection. In order to emphasize the variability in the effects of differing arts therapies on COVID-19-induced psychiatric disturbances, the sourced literature was systematically categorized and scrutinized based on distinct modalities.

**Results:**

Out of the 7,250 sourced articles, 16 satisfied the inclusion conditions. The therapies were predominantly meditation (*n* = 7), supplemented by individual studies on color therapy (*n* = 3), music therapy (*n* = 2), and single studies on horticultural therapy, dance therapy, mindfulness and music therapy, and yoga and music therapy (*n* = 4 collectively). These various forms of arts therapies had a positive short to medium-term impact on the mental health of COVID-19 patients. Besides improving patients' physical and mental health, these therapies can also be employed to mitigate mental health issues among healthcare professionals.

**Conclusion:**

The COVID-19 pandemic has profound and long-lasting implications for public mental health. Diverse forms of arts therapies are potentially effective in addressing related psychiatric symptoms. The integration of artificial intelligence might further enhance the efficacy and scalability of arts therapies in future implementations.

## 1 Introduction

As of August 13, 2023, the cumulative number of COVID-19 cases and related variants reached 769 million globally. The global death amounted to 6.9 million, accounting for 0.08% of the global population (1). The COVID-19 pandemic has far-reaching impacts on various societal sectors, generates considerable stress among healthcare workers (2, 3), and induces significant emotional strain on the general population due to the extensive infection rates and associated mortality (4–6). This considerable burden leads to psychological and mental health problems, emanating not only from the physical symptoms of the disease but also from various stressors (7, 8).

The COVID-19 pandemic, as reported by the World Health Organization, has given rise to multitudinous stressors, which notably include economic hardships, disease prevalence, workforce reductions, and rigid governmental policies (9–11). Such stressors significantly exacerbate mental health issues, leading to a broad spectrum of psychological disorders. If these emerging psychological health challenges are not addressed promptly, they could escalate into severe conditions such as anxiety, depression, intense stress, profound sorrow, suicidal ideations, and feelings of isolation. These conditions may subsequently induce more severe disorders like eating disorders, obsessive-compulsive disorder, and traumatic stress responses, further weakening the body's immune responses (12). Affected demographics encompass individuals with pre-existing mental health conditions, women, parents, the older adult, racial minorities, children, college students, and military personnel, with those having a history of psychological disorders or predisposition to isolation exhibiting heightened vulnerability (13, 14).

In the context of recurring COVID-19 outbreaks and the concomitant governmental policies and quarantines, there was an increase in psychiatric symptoms across both infected and uninfected populations, which was further aggravated by a shortage of specialized medical facilities and the lack of immediate treatment options (15). This situation accentuated the need for practical, economical therapeutic strategies that extend beyond conventional pharmaceutical treatments. These strategies ought to effectively manage these symptoms and thus improve people's quality of life. Therefore, the time is ripe to consider the potential of alternative therapeutic interventions, such as art therapy, a well-established method, to address the psychological health issues resulting from COVID-19 and Post-Acute COVID-19 Syndrome.

This review primarily focused on utilizing arts therapies to treat psychological disorders in COVID-19 patients. According to the British Association for Art Therapy (BAAT), art therapy is “a form of psychotherapy that uses art media as its primary mode of expression and communication” to support individuals in distress (16). According to the American Art Therapy Association (AATA), Through integrative methods, art therapy engages the mind, body, and spirit in ways that are distinct from verbal articulation alone. Art Therapy offers numerous sub-disciplines to combat distinct mental ailments, frequently employing a variety of artistic mediums in both group and individual environments (17). The current review primarily concerns itself with the following research issues:

Which literature can be incorporated into the discussion about the effectiveness of arts therapies for the mental health problems of COVID-19 patients?

What distinct sub-therapies can effectively address specific mental health issues?

How can the efficiency of arts therapies be elevated in the future for large-scale populations?

review aims to contribute to the respective literature by identifying the mechanisms of action, consolidating the consensus on the effectiveness of arts therapies, discussing persisting issues in research design, and suggesting future steps for advancing the application of arts therapies in treating mental disorders (18, 19).

## 2 Materials and methods

### 2.1 Searching literature about the arts therapies

A methodical search was performed on PubMed, Cochrane Library databases, Embase, Web of Science, and CNKI databases to review the literature and identify relevant articles comprehensively. Utilizing MeSH tags such as “art therapy,” “music therapy,” “dance therapy,” “color therapy,” “play therapy,” “drama therapy,” and “video games”, these keywords were combined with terms like “COVID-19,” “Post-Acute COVID-19 Syndrome,” “mental disorder,” “autistic disorder,”, “schizophrenia,” “depression,” “bipolar disorder,” “anxiety,” and “post-traumatic stress disorder (PTSD).” The timeframe for this literature search was limited to articles published from January 2000 to July 2023, as further expounded in Table 1. Inclusion criteria mandated the incorporation of creative art therapies for mental illnesses by the categories delineated above. Any articles that were not available in the English language were consequently excluded from consideration.

**Table 1:** Search strategies for English databases or Chinese databases.

**Number**	**Search terms**
#1	Art therapy [MeSH]
#2	Color therapy [MeSH]
#3	Music therapy [MeSH]
#4	Play therapy [MeSH]
#5	Psychodrama [MeSH]
#6	Dance therapy [MeSH]
#7	Video games [MeSH]
#8	#1 OR #2 OR #3 OR #4 OR #5 OR #6 OR #7
#9	COVID-19 [MeSH]
#10	Post-acute COVID-19 syndrome [MeSH]
#11	Mental disorders [MeSH]
#12	Autistic disorder [MeSH]
#13	Anxiety [MeSH]
#14	Depression [MeSH]
#15	PTSD [MeSH]
#16	Bipolar [MeSH]
#17	Schizophrenia [MeSH]
#18	#9 OR #10 OR #11 OR #12 OR #13 OR #14 OR #15 OR #16 OR #17
#19	#8 AND #18
#20	Yishu zhiliao (Art therapy)
#21	Huihua liaofa (Color therapy)
#22	Yinyue liaofa (Music therapy)
#23	Youxi liaofa (Play therapy)
#24	Xiju liaofa (Psychodrama)
#25	Wudao liaofa (Dance therapy)
#26	Youxi liaofa (Video games)
#26	#20 OR #21 OR #22 OR #23 OR #24 OR #25 OR #26
#27	#18 keywords translated into Chinese
#28	#26 AND #27

The search method was derived from the PubMed database and applied to additional databases. This formula was used consistently across all databases: (“Art Therapy”[Mesh]) OR “Music Therapy”[Mesh]) OR “Color Therapy”[Mesh]) OR “Play Therapy”[Mesh]) OR “Dance Therapy”[Mesh]) OR “Psychodrama”[Mesh]) OR “Video Games”[Mesh]) AND “COVID-19”[Mesh]) OR “Post-Acute COVID-19 Syndrome”[Mesh]) AND (“Mental Disorders”[Mesh]) OR “Autistic Disorder”[Mesh]) OR “Anxiety”[Mesh]) OR (“Depression”[Mesh] OR “Depressive Disorder”[Mesh])) OR “Stress Disorders, Post-Traumatic”[Mesh]) OR “Bipolar Disorder”[Mesh]) OR “Schizophrenia”[Mesh]).

**Figure 1:**
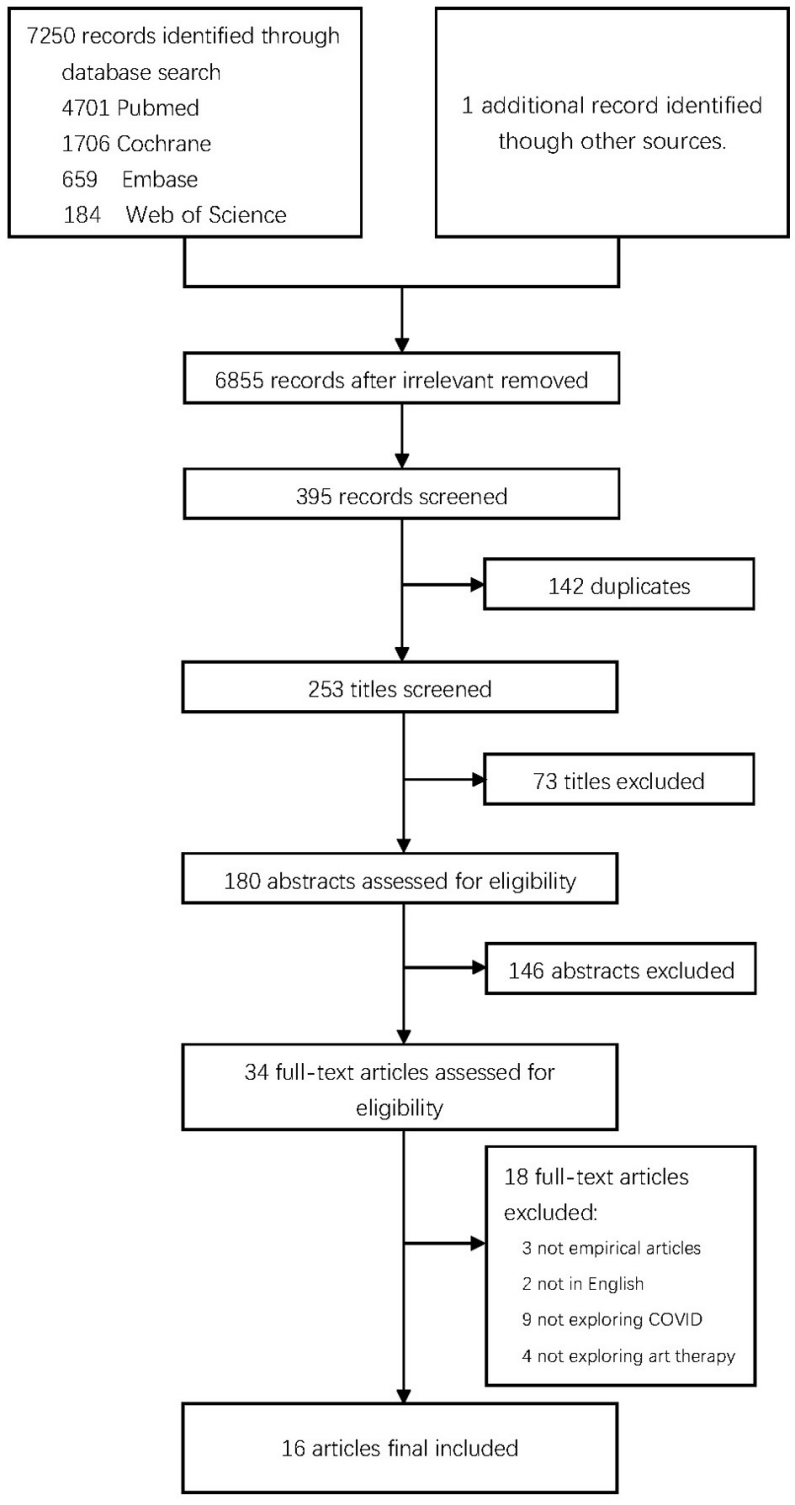
Flow diagram for the included and excluded articles.

A total of 7,250 citations conforming to the search criteria were identified, from which 34 full texts underwent examination (refer to Table 1; Figure 1).

The framework illustrated in Figure 1 outlines the inclusion and exclusion criteria for the study. Articles were rigorously screened based on these criteria, posed as three questions: 1. Does the article's content pertain to COVID-19, post-COVID-19, and arts therapies or its sub-therapies? 2. Does the article provide relevant data to validate the application of arts therapies? 3. Does the article possess significant value? The first two questions guided the initial screening and subsequent literature review.

The third question was instrumental in determining the three exclusion criteria: 1. Insufficient value in the literature. 2. non-English literature. 3. Literature classified under “gray literature.” During the literature screening process, it became apparent that certain interventions were defined ambiguously, thereby complicating the differentiation between art psychotherapy applications and the mere utilization of art for recreational purposes or the acquisition of artistic skills. By adhering to this criterion, studies were assessed exclusively for their exploration of arts therapies, ensuring the elimination of any ambiguities in intervention classification.

We included papers relating to the MATISSE trial identified in the search to review them in light of the more exhaustive research (19, 20).

## 3 Results

### 3.1 Method of quality appraisal

Two independent reviewers, LXX and JW, were involved during the screening, eligibility, and inclusion review process. LXX was responsible for downloading and reviewing the screened articles, excluding irrelevant literature. The relevant literature was then forwarded to JW for eligibility review. JW assessed the eligibility of the literature to be included in the analyses. The literature that met the eligibility criteria underwent a double inspection by both LXX and JW.

The 16 selected articles delivered precise data and research content. Their quality was evaluated by a multimethod framework established based on standardized criteria (21). This framework relied on multiple strategies to extract and analyze data and content from the pertinent references (22), incorporating qualitative (23), multi-method (24), target audience, and RCT strategies (25). The analysis method was updated in light of two papers (26, 27); in adherence to the Center for Review and Dissemination's recommendations, an extra researcher was included to solidify the framework (28). The finalized framework encompassed four primary quality categories, with each article being assessed based on these criteria: Validate, Suitability, Therapeutic, and Consistency. Scores were assigned to each category, and the average score of all four categories determined the article's overall quality rating and robustness as excellent, good, moderate, or poor. To resolve discrepancies in ratings, WJ and independent researchers conducted a calibration of quality ratings on samples from 16 studies (see Table 2); we used study design by PICO[S] to identify RCT data in these 16 studies (see Table 3).

**Table 2:** Record of citation score.

	**Validate**	**Suitability**	**Therapeutic**	**Consistency**	**Overall score**
**Multimethod study**					
William et al. (29)	4	3	3	3	Moderate (3.25)
Zildzic et al. (30)	3	2	3	4	Moderate (3)
Vinciguerra and Federico (31)	3	3	3	3	Moderate (3)
Vajpeyee et al. (32)	4	2	4	3	Moderate (3.25)
Yi et al. (33)	5	4	3	4	Excellent (4)
**Quantitative study**					
Calamassi et al. (34)	4	2	3	3	Moderate (3)
Yildirim et al. (35)	4	3	4	4	Good (3.75)
Khademi et al. (36)	5	4	4	4	Excellent (4.25)
Shao (37)	3	3	4	3	Moderate (3.25)
Thimmapuram et al. (38)	5	4	3	3	Good (3.75)
**Report study**					
Matiz et al. (39)	5	4	4	4	Excellent (4.25)
Jiménez et al. (40)	4	4	3	4	Good (3.75)
Desai et al. (41)	4	4	3	3	Good (3.5)
Priyanka and Rasania (42)	4	5	3	4	Excellent (4)
Bhuiyan et al. (43)	4	3	4	3	Good (3.5)
Renzi et al. (44)	3	2	2	2	Poor (2.25)

**Table 3:** Principal characteristics of all included RCTs in this review.

**References**	**ID**	**Design**	**Sample size (T/C)**	**Outcomes measure**	**Treatment group**		**Control group**
					**Intervention**	**Procedure times**	
Calamassi et al. (34)	31 (1)	RCT	18/19/17^*^	STAI/HR/RR/SBP/DBP	MT	1	TT
Yildirim et al. (35)	33 (2)	RCT	52/52	STAI/WRSS/PWBS	MT	1	TT
Khademi et al. (36)	37 (3)	RCT	35/35	STAI	MC	6	TT
Shao (37)	43 (4)	RCT	32/30	SCL-90/LSS/ADS/MPR	DT	7	TT
Thimmapuram et al. (38)	44 (5)	RCT	77/78	UCLA/PSQI	HM	4	TT

TT, traditional therapy; MT, Music Therapy; DT, Dance Therapy; HM, Heartfulness Meditation; STAI, State-Trait Anxiety Inventory; HR, Heart Rate; RR, Respiratory Rate; SBP, Systolic Blood Pressure; DBP, Diastolic Blood Pressure; WRSS, Work-Related Strain Scale; PWBS, Psychological Well-Being Scale; MC, Mandala Coloring; SCL-90, Symptom Checklist 90; LSS, Life Satisfaction Scale; ADS, Anxiety and Depression Scale; MPR, Measurement of psychological resilience; UCLA, UCLA loneliness scale; PSQI, Pittsburgh sleep quality index.

^*^ID-31(1) group has split into 3 different intervention therapy which is *N* = 18/19/17, the whole count number is 54.

LXX extracted data from each study using a grid that summarized information relevant to the goals of the review (Table 4). JW randomly selected the papers to double-check to avoid missing or inaccurate data.

**Table 4:** Record of citation analyses and full texts reviewed.

**References**	**Types**	**Nation**	**Method**	**Study aims**	**Participants**	**Procedure**	**Analysis**	**Results of art therapy**
Calamassi et al. (34)	RCT	Italy	Music therapy	To analyze the impact Hertz of music can provide a better effect on COVID-19 relevant mental disorders	54 participants, 32 females (59.3%); mean age of 39.64 years (SD ± 9.94); the total measurements performed were 83	Set 54 participants randomly into three groups; three groups are testing different Hertz music.	SPSS Statistical Software version 17^®^ and GraphPad Prism 9^®^. The Wilcoxon Signed Rank Test (dependent samples) and the *t*-test (paired samples).	432 Hz music can reduce the stress and mental anxiety of COVID-19 emergency nurses.
Bushell et al. (29)	Review	USA	Meditation	To analyze the effect of Yoga and meditation as performance art on COVID-19 relevant mental symptoms	Non-participants were briefed to organize different data.	Compare different backgrounds of the pandemic with performance art therapy.	Data comparison and reference extraction to organize a statement with content.	Preliminary evidence for possible forms of immune system enhancement accompanying the practice of forms of meditation and yoga.
Yildirim et al. (35)	RCT	Turkey	Mindfulness and Music Therapy	To analyze the effect of Breathing and music therapy on COVID-19 relevant nurses.	A total of 104 participants split into two groups: 52 in intervention and 52 in control	Half-hour test with two groups to obtain the intervention and control group data.	Mann–Whitney *U*-test and χ2 test were utilized in the identification of the statistically significant differences between groups, and the Wilcoxon signed rank test was utilized.	Mindfulness-based breathing and music therapy reduced nurses' stress and work-related strain levels and enhanced their psychological wellbeing.
Matiz et al. (39)	Self-report	Italy	Meditation	To analyze the impact of mindfulness meditation on mental health with female teachers in Italy	A total of 67 participants were further split into a low-resilience (LR, *n* = 32) and a high-resilience (HR, *n* = 26) group.	Eight weeks relevant training program to test different groups which able to receive a better result on mental disorders.	The analyses were performed with the free software environment R, version 3.6.3 (R Foundation for Statistical Computing, Vienna, Austria).	Participants actively engaged daily in the practice of meditation to protect and promote their mental health.
Jiménez et al. (40)	Self-report	Spain	Meditation	To explore the impact of confinement due to COVID-19 on the mental and emotional health of adult Spanish-speaking residents of Spain.	A total of 412 adults from 63 Spanish provinces, with a mean age of 40.48 (SD = 10.79), participated in the survey out of a total of 420 (finalization rate: 98.09%).	This paper collected survey data with the method of DASS-21, IES, and SCS scales. Data extraction as proof to procedure the result related to study aims.	All the analyses were performed with SPSS version 26.0 (IBM Corp., Armonk, NY, USA) and The descriptive results of the quantitative variables were reported with mean and standard deviation.	The findings of this study could be used for psychological interventions to Improve mental health and coping with confinement during the COVID-19 epidemic.
Desai et al. (41)	Self-report	USA	Meditation	To investigate whether using a virtual heart-based meditation program is associated with improved stress levels and quality of sleep among participants from the general population during the COVID-19 pandemic.	A total of 63 participants were enrolled in the study, of which 36 (57%) completed the entire 8 weeks of the heartfulness meditation program.	An 8-week virtually conducted heartfulness. meditation program in a prospective pre-post single-arm intervention study	To evaluate their perceived stress and sleep quality using measurement tools with well-established reliability and validity, such as the Perceived Stress Scale (PSS) and the Pittsburgh Sleep Quality Index (PSQI)	Statistical improvements in perceived stress score and sleep quality index in participants undergoing a virtual heartfulness meditation program.
Khademi et al. (36)	RCT	Iran	Color Therapy	To investigate the effect of Mandala coloring on the anxiety of hospitalized COVID-19 patients.	A total of 70 patients were hospitalized with COVID-19 in the internal medicine and infectious disease wards.	A randomized controlled clinical trial performed from May 20, 2020, to November 30, 2020, on 70 patients hospitalized with COVID-19	The data were analyzed using SPSS (version 25) software. Quantitative variables were described using mean and standard deviation, and qualitative variables were presented by frequency and percentage.	Mandala coloring was effective in reducing measured anxiety in patients hospitalized due to COVID-19.
Priyanka and Rasania (42)	Self-report	India	Meditation	To explore and investigate the effect of yoga and meditation can help in alleviating mental. Stress and improving psychological wellbeing.	A total of 1,112 subjects gave consent and completed the study questionnaire. Of them, 861 (77.4%) responses were obtained in English, and 251 (22.6%) were in Hindi questionnaire.	A community-based online cross-sectional study involving the adult general population. Data collection was done by using a Google form link that was circulated via online platforms.	The data were analyzed using Microsoft Excel and SPSS version 22. Qualitative data were expressed in proportions or percentages, and quantitative data were expressed in mean and standard deviation. The chi-square test was used to check the association between various factors and mental wellbeing.	The practice of yoga and meditation, preferably both of them, is associated with a higher level of mental wellbeing during the COVID-19 pandemic.
Zildzic et al. (30)	Review	Bosnia and Herzegovina	Horticultural therapy	To evaluate the impact of non-pharmacological measures such as stress and sleep control (with different measures against the negative effects of anxiety and depression on the mental state) and the possible positive impact of “forest bathing” on improving the immune response to the virus and its consequences.	N/A	Available evidence-based studies on ways to combat stress and the effect of the proposed measures on human mental health and the immune system were analyzed.	From those studies, recommended measures have been registered, which refer to stress and sleep control, diet and eating habits, staying in nature (“forest bathing”, gardening), virtual communication, and meditation (mindfulness practice).	Non-pharmacological measures such as stress and sleep control, spending time in nature, a healthy diet, and physical activity may improve the immune response to COVID-19.
Bhuiyan et al. (43)	Self-report	USA	Meditation mobile meditation app.	To explore associations between rural or urban status, psychological outcomes, and physical activity among users of a (7,704/8,392, 91.8%)	A total of participants *(N* = 8,392) were mostly female (7,041/8,392, 83.9%), non-Hispanic (7,855/8,392, 93.6%), and White	A secondary analysis of a national survey conducted among subscribers to the meditation app Calm.	All statistical analyses were performed using SPSS, version 26.0 (IBM Corp), with significance inferred at *P* < 0.05.	No found associations between rural or urban status and psychological outcomes (stress, depression, and anxiety), pre–COVID-19 and current physical activity, or perceived effects of COVID-19 on stress, mental health, and physical activity among users of a meditation app.
Renzi et al. (44)	Case-report	Italy	Color Therapy	A study to report art method on older adult who isolation during the COVID-19 period	An older adult woman (age: 77 years old; Mini-Mental State Examination score: 30; Geriatric Depression Scale score: 6) who spent 3 months in isolation in her room in our nursing home as a prevention strategy during COVID-19 pandemic	An older adult woman participant drew three paints and communicated with a psychologist to identify her mental status, further recording as a case report to show the impact on patients.	Psychological counseling session between the participant and psychologist to analyze what mood she has changed during the Lockdown period.	Evidence from multiple studies has shown that drawing offers short-term mood benefits for adults.
Vinciguerra and Federico (31)	Review	Italy	Music Therapy	To evaluate the therapeutic effects of the MBIs (music-based interventions) and to discuss the feasibility, accessibility, and future implementation of these new NMT (new music therapy) approaches.	N/A	Selected all the articles registered in the Web of Knowledge, PubMed, Google Scholar, and ScienceDirect from March 2020 to November 2021 concerning tele-NMT during the COVID-19 outbreak, collecting the same examples and experiences.	Extract the content and results from the other references and organize the discussion of the effect on COVID-19 patients with art therapy.	With the advent of COVID-19, several music-based interventions (MBIs) have been adapted from “in-person.” To a “remote and virtual” mode (through telemedicine).
Shao (37)	RCT	China	Dance Therapy	To investigate the intervention effect of dance therapy based on the Satir Model on the mental health of adolescents with depression during the COVID-19 epidemic.	A total of 62 adolescents finally completed the experiment. contents	Using Symptom Checklist 90 and randomly divided into two groups according to the matching of male and female participants, the experiment group had 32 members, and the control group had 30 members.	All the data in this study were processed using SPSS 17.0 statistical software. The comparison between groups was conducted using an independent sample *t*-test, and the comparison within the group was conducted using a paired sample *t*-test. The difference is statistically significant at *p* > 0.05.	The results showed that this comprehensive group psychological counseling can effectively improve the mental health level of adolescents alleviates their anxiety and depression, increases their life satisfaction, and promotes their psychological resilience level.
Thimmapuramet al. (38)	RCT	USA	Meditation	To investigate if a brief, virtual, heart-based meditation program via audio relaxation techniques through a heartfulness trainer leads to measurable changes in the improvement of sleep and perception of loneliness in physicians and advanced practice providers.	Out of 1,535 eligible participants who were surveyed, 155 enrolled in the study.	Physicians and advanced practice providers were randomly assigned to receive either a daily heartfulness Meditation program or no intervention (control group) in a prospective 4-week randomized control study design. UCLA loneliness and PSQI scores were collected at baseline and after the program duration of 4 weeks.	Data were summarized using frequencies and means/medians and reported using per-protocol analysis. Changes in loneliness and PSQI scores were analyzed by paired t-tests, and an α < 0.05 was considered statistically significant. Pearson correlation test was carried out. Statistics were calculated using SPSS v.24 (IBM, Armonk, NY).	The current research Is one of the first attempts to assess loneliness and sleep problems among physicians and advanced practice providers during the COVID-19 pandemic in the US. heartfulness meditation appears to provide an improvement in the perception of loneliness and sleep quality.
Vajpeyee et al. (32)	Review	India	Yoga and Music Therapy	To investigate the impact of Yoga and Music Intervention on anxiety, stress, and depression levels of healthcare workers during the COVID-19 outbreak.	Of all 209 participants, 105 (50.23%) had symptoms of depression (35.88%), anxiety (40.19), and stress (34.92%) alone or in combination.	To assess the psychological responses of 240 healthcare workers during the COVID-19 outbreak. We used Yoga and Music Intervention in normal and abnormal subjects based on the Depression Anxiety and Stress Scale-42 (DASS-42).	Statistical analysis was performed using Microsoft Excel. The mean is calculated, and for hypothesis testing, a student *t*-test is applied to compare the mean before and after the intervention on both controlled and uncontrolled groups.	The significance of easily available, simple, inexpensive, safe non-pharmacological interventions like Yoga and Music therapy is to overcome stress, anxiety, and depression in present times.
Yi et al. (33)	Review	China	Color Therapy	The positive role of interface visual design in digital safety education was verified by taking COVID-19 prevention and control knowledge as the content of public health safety education	252 college students (119 men and 133 women) were recruited as participants from local universities in southern China via a purposive sampling method.	A 3 (interface emotions) _ 2 (interface layouts) two-factor experimental design was adopted, and 6 interfaces were designed, where the dependent variables included participants' understanding, course evaluation, and system usability score.	Utilized the Two-Way ANOVA for analyzing relevant experimental data with IBM SPSS (version 24). For significantly different factors, a *post-hoc* test was conducted.	Visual design indeed has an impact on the effectiveness of learning about COVID-19 protection.

N/A means no participants.

In this review, we presented a descriptive synthesis of qualitative and methodological aspects of the papers considered in five quality categories.

### 3.2 Treatment method

The Taiwan Association for Art Therapy (TATA) believes that the expression of art therapy often utilizes mental images for reflection. That art involves the client applying their perceptions and senses. In contrast, the Hong Kong Association of Art Therapists (HKAAT) believes that Art Therapy is about using art as a communication tool. Through the therapeutic relationship, emotional, psychosocial, and developmental needs are addressed to effect lasting change. In summary, arts therapies' core lies in the driving force of art, which itself is diverse in its forms of expression; arts therapies encompass a variety of concepts, rendering it a complex therapeutic approach due to its diverse types (45). Commonly associated with psychology and psychiatry (46–50), arts therapies include music therapy (32, 51–53), mandala-color therapy (36, 54), creative art therapy (45, 55, 56), dance therapy (53, 57), play or game therapy (58–61), and meditation or yoga therapy (29, 39, 41, 42, 62, 63). These art therapies comprise proactive approaches—such as mandala coloring, dance therapy, and meditation therapy—where patients focus on the activity to divert negative mental attention. Conversely, reactive approaches like music therapy or art gallery-based projects entail patients receiving positive stimuli from external sources to benefit their mental health (64), the article presents a tabular representation that effectively illustrates the outcomes derived from the literature about the seven distinct therapeutic modalities (see Table 5).

**Table 5:** Comparative analysis of results across modalities.

**Method**	**References**	**Types**	**Results**
Music therapy	Calamassi et al. (34)	RCT	432 Hz music can reduce the stress and mental anxiety of COVID-19 emergency nurses.
	Vinciguerra and Federico (31)	Review	With the advent of COVID-19, several music-based interventions (MBIs) have been adapted from “in-person.” To a “remote and virtual” mode (through telemedicine).
Meditation	Bushell et al. (29)	Review	Preliminary evidence for possible forms of immune system enhancement accompanying the practice of forms of meditation and yoga.
	Matiz et al. (39)	Self-report	Participants actively engaged daily in the practice of meditation to protect and promote their mental health.
	Jiménez et al. (40)	Self-report	The findings of this study could be used for psychological interventions to Improve mental health and coping with confinement during the COVID-19 epidemic.
	Desai et al. (41)	Self-report	Statistical improvements in perceived stress score and sleep quality index in participants undergoing a virtual heartfulness meditation program.
	Priyanka and Rasania (42)	Self-report	The practice of yoga and meditation, preferably both of them, is associated with a higher level of mental wellbeing during the COVID-19 pandemic.
	Bhuiyan et al. (43)	Self-report	No associations between rural or urban status and psychological outcomes (stress, depression, and anxiety), pre–COVID−19 and current physical activity, or perceived effects of COVID-19 on stress, mental health, and physical activity among users of meditation app.
	Thimmapuram et al. (38)	RCT	The current research Is one of the first attempts to assess loneliness and sleep problems among physicians and advanced practice providers during the COVID-19 pandemic in the US. heartfulness meditation appears to provide an improvement in the perception of loneliness and sleep quality.
Color therapy	Khademi et al. (36)	RCT	Mandala coloring was effective in reducing measured anxiety in patients hospitalized due to COVID-19.
	Renzi et al. (44)	Case-report	Evidence from multiple studies has shown that drawing offers short-term mood benefits for adults.
	Yi et al. (33)	Review	Visual design indeed has an impact on the effectiveness of learning about COVID-19 protection.
Horticultural therapy	Zildzic et al. (30)	Review	Non-pharmacological measures such as stress and sleep control, spending time in nature, a healthy diet, and physical activity may improve the immune response to COVID-19.
Dance therapy	Shao (37)	RCT	The results showed that this comprehensive group psychological counseling can effectively improve the mental health level of adolescents alleviate their anxiety and depression, increase their life satisfaction, and promote their psychological resilience level.
Mindfulness and music therapy	Yildirim et al. (35)	RCT	Mindfulness-based breathing and music therapy reduced nurses' stress and work-related strain levels and enhanced their psychological wellbeing.
Yoga and music therapy	Vajpeyee et al. (32)	Review	The significance of easily available, simple, inexpensive, safe non-pharmacological interventions like Yoga and Music therapy is to overcome stress, anxiety, and depression in present times.

Patterson and colleagues found that art therapy is typically divided into two methods: 94.4% of individuals receive art therapy from therapists, and 70.4% engage in group-based therapy with therapists (65). Art therapy treatments can be categorized as open-ended (47, 50, 66) or closed-loop (46). For controlled trials to establish the efficacy of arts therapies, participants must follow a stringent program directed by an art therapist. Individuals with severe symptoms typically necessitate lengthier interventions for optimum outcomes (47, 67, 68).

Through the examination of various therapeutic methodologies, it is evident that many individuals have encountered isolation within the framework of COVID-19 infection. Moreover, the implementation of diverse isolation policies by different nations, some involving prolonged periods of closure and seclusion, has resulted in concealed mental health issues among individuals residing in confined and inaccessible environments during isolation. These individuals often exhibit psychological sensitivity, irritability, and anxiety. However, it has been observed that arts therapy interventions can effectively mitigate or eliminate these psychological states and problems, thereby averting their progression into severe conditions over time. Art therapy has effectively reduced or eliminated certain psychological disorders and problems, mitigating the risk of these issues progressing into more severe states.

In summary, several arts therapy modalities vary in their respective intervention durations. As an illustration, engaging in activities such as listening to music and practicing meditation does not necessitate intricate apparatus or a particular setting. These therapeutic modalities can be employed amidst periods of illness and seclusion to modulate one's emotional state effectively.

### 3.3 The therapeutic of arts therapies

Five quantitative studies, including four randomized controlled trials (RCTs), assessed arts therapies' efficacy and therapeutic effects. The mental status of patients who received arts therapies over a specific period was compared to a control group undergoing traditional medication (34–38). Two studies focused on healthcare workers such as nurses involved in treating COVID-19 patients (34, 35), while the remaining three targeted patients with COVID-19 (36–38). These studies used various widely accepted therapeutic approaches to primarily measure quality of life outcomes and therapeutic effects, including art therapy (45), music therapy (34, 35), color therapy (36), dance therapy (37), and meditation therapy (38).

Table 3 presents the PICO(S) framework, which illustrates the utilization of music therapy, dance therapy, mandala therapy, and meditation in addressing mental health issues among individuals affected by COVID-19. The results of five randomized controlled trials (RCTs) indicate that these therapeutic interventions have yielded positive outcomes. Notably, the scope of these outcomes extends beyond the patients themselves to include healthcare practitioners. The literature about these RCTs suggests that various forms of arts therapies have been employed with diverse populations. In these interventions, subjects were exposed to rigorous programs by medical professionals or art therapists.

The results suggested that ~6–12 days of arts therapies intervention can ameliorate mental symptoms, including anxiety, depression, and insomnia. Although the severity of various disorders differs from the control group, music, and color therapies typically enhance mild anxiety in patients or nurses swiftly. Dance and meditation therapies predominantly aim at moderate symptoms such as mild depression and sleep disturbances. Results demonstrate that these mid-level mental disorders usually necessitate consistent, long-term treatment to show comparable positive outcomes to the control group.

Preliminary conclusions from other studies imply that individuals with severe mental illness consequent to COVID-19 may not be optimally suited for exclusive arts therapies due to the many physiological symptoms associated with the virus. Persistent post-COVID-19 symptoms fluctuate in intensity, during which patients typically exhibit mental and physical symptoms (69). Research findings underscore the value of arts therapies as a supplementary approach for critically ill mental health patients within a clinical setting. Patients with schizophrenia, bipolar depression, or suicidal tendencies often necessitate auxiliary interventions like medications or electroconvulsive therapy (70–73).

### 3.4 Outcome measures

According to BAAT, AATA, TATA, and HKAAT, which collectively state that the drive for art is the biggest centerpiece in arts therapies, arts therapies serve as a bridge connecting patients to their inner worlds, with art therapists and the artistic process acting as catalysts for uncovering deep-seated emotional issues. Nevertheless, study findings indicate variability in results, frequently influenced by factors including the stage of professional treatment, prime physiological state, and acquaintance with art therapists and techniques. Therefore, precisely evaluating the impact of arts therapies on patients with mental disorders and potentially associated groups (e.g., individuals experiencing prolonged quarantine) remains a challenge (74, 75). Such a population faces a higher risk of mental illness due to enforced quarantine policies instead of voluntary compliance (76, 77). Large-scale events are often unpredictable and unplanned, sometimes resulting in insufficient resources during quarantine.

The primary outcome measures In three RCTs involving patients (36–38) may not fully encompass potential group dynamics or observations from individuals in quarantine with COVID-19-positive cases at risk of infection. These individuals often experience anxiety, nervousness, and restlessness (11); prompt arts therapies intervention could potentially inhibit further deterioration. This finding aligns with psychotherapy's emphasis on alleviating distress and fostering coping mechanisms rather than simply reducing symptoms (78). Secondary outcome measures in these RCTs included aspects such as social functioning, wellbeing, mentalization, and self-efficacy (79), which require a more comprehensive study. Furthermore, self-confidence and intra- and interpersonal connectedness are proposed as potential outcome measures for arts therapies.

### 3.5 Intervention design

**Figure 2:**
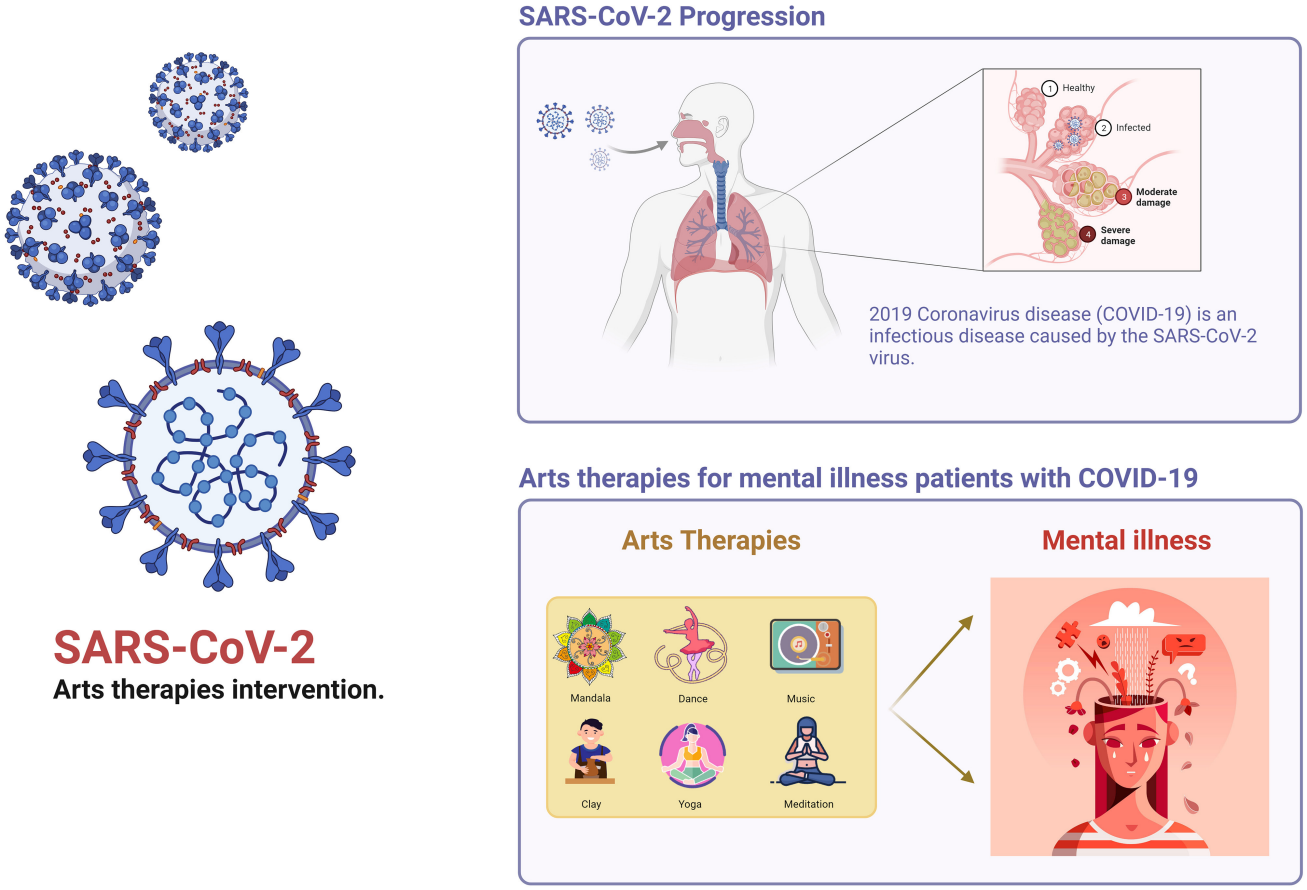
Diagram of the relationship between arts therapies and COVID-19 mental illness.

Arts therapies can be a versatile supplementary approach for patients with different types of mental disorders (refer to Figure 2). Therefore, the statistical analysis of risk and mental status changes should be personalized according to the diverse participant groups. Future RCTs could be enhanced by stratifying the research populations based on the subtleties of mental health issues related to COVID-19. Recognizing distinct characteristics of patients and potential patients is crucial for subsequent studies. During mandatory quarantine, many individuals may experience changes in their typical mental behavior (11, 75, 80), underscoring the need for early intervention.

Art therapy, a non-invasive, cost-effective method with no associated side effects, can be administered early to potential patients in controlled quarantine. Employing techniques such as music intervention, mandala-color intervention, and web-based guided meditation developed by art therapists may yield benefits. Simultaneously, evaluations and statistics following MATISSE guidelines (46, 81) can be used to assess individuals' mental status during quarantine and for 7–14 days afterward, determining the potential reduction in the incidence of mental illness due to arts therapies.

The inclusion of regular follow-up periods in research may elucidate the long-term effects of arts therapies, which is not evidenced in the reviewed studies (23). In the MATISSE framework, transient improvements from arts therapies could be missed (16); hence, diligent data documentation—particularly at the onset, midpoint, and closure of the intervention—is vital to measure changes accurately.

## 4 Discussion

Amid the detrimental effects of the COVID-19 pandemic, existing literature acknowledges the far-reaching impact of the virus beyond physical health, with significant implications for global mental health. This reality necessitates urgent, in-depth research into therapeutic responses to manage pandemics' psychological repercussions. Enhanced understanding of psychological burdens across various populations would significantly facilitate this effort (2, 3). Present research emphasizes pandemics' impact on mental health (4–6), the manifestation of complex stressors such as economic hardship, disease epidemics, labor force reductions, and stringent government policies exacerbating global mental health crises (9–11). Thus, developing non-pharmacological treatments for mild to moderate psychological and mental health issues arises as a key research area.

To further alleviate this crisis, current research strives to broaden our understanding of psychiatric symptoms displayed by COVID-19-affected and unaffected populations, evaluating the effectiveness of different forms of art therapy in targeting mental illnesses. The findings underscore the urgent need to develop efficient and cost-effective treatment strategies, including variably time-intensive art therapy forms alongside traditional medication (15). A comparative analysis with prior studies presents a crucial evolution: While most studies have primarily explored health issues from a biomedical perspective, the inclusion of 16 art therapy papers illuminates the feasibility of alternative therapies for intervening mild-to-moderate psychological and mental health issues. This shift in focus, especially amidst COVID-19's substantial pressure on global mental health resources, is of paramount importance.

This review seeks to extend this newly adopted perspective, exploring art therapy's role and potential in managing mental health issues associated with COVID-19 and its sequelae. Consequently, this paper's narrative diverges from conventional treatment protocols, recognizing alternative therapeutic interventions' potential value.

Diletta et al. assert the necessity of artistic interventions for healthcare professionals in music therapy, suggesting telemedicine's applicability in the current context (31, 34). In meditation therapy, involving 11,273 of the 16 papers' subjects, William posits meditation and yoga can enhance the body's immune system to combat viruses (29). Desai et al. suggests meditation also improves sleep quality (41). While Bhuiyan reported no difference between rural or urban status concerning psychological issues (43), the majority of included meditation literature is based on quantitative questionnaire statistics, indicating a need for more RCTs to understand meditation's role across different ages and cultures. Khademi, Renzi, and Yi used mandala therapy and painting therapy, noting their dual capacity to alleviate patients' anxiety as tools for evaluating patients' mental states (33, 36, 44). Zildzic's et al. study deems horticultural therapy effective in ameliorating sleep and stress issues, as well as immune system problems during the COVID-19 pandemic (30). Lastly, Shao's study found dance therapy beneficial in managing adolescent anxiety and depression (37). However, traditional dance may not be suitable for all, particularly middle-aged and older adult individuals or post-COVID-19 patients. Manisha's proposal of a music-and-yoga combination demonstrates better efficacy for these demographics (32), indicating the need for further research into non-pharmacological alternative therapies customized for different populations. Examples for future study may be Qigong and Tai Chi.

Through PICO(S), we conducted a structured data extraction from the five included RCTs, encompassing 445 subjects. Three RCTs used the STAI scale for anxiety, with the remaining using the WRSS for work stress and PWBS for mental health status (34–36). Calamassi's RCT also measured heart rate, respiratory rate, and blood pressure to validate meditation's effects (34), whereas other RCTs used varying scales to evaluate life satisfaction, anxiety, depression, loneliness, sleep quality, and psychological wellbeing (37, 38). All RCTs effectively evaluated the effectiveness of different art therapy subtypes, though underscored the need to balance the patient-to-practitioner ratio.

Collectively, these studies underscore the growing promise of treatments for COVID-19-related mental health disorders. Given the broad COVID-19 patient base, a large number of people suffer from mental health issues. Factors such as isolation and closure render individuals psychologically vulnerable, making teletherapy an invaluable asset. However, the number of psychologists is inadequate to maintain a one-to-one counseling ratio. In this context, we propose a hypothesis: Could artificial intelligence assist psychologists and extend the reach of art therapy?

As the number of patients with NCCP-associated psychiatric disorders increases, traditional art therapies such as painting and music face limitations. The technical complexity and time-consuming nature of traditional painting techniques make it challenging to manage large numbers of patients simultaneously. Similarly, music therapy struggles with selecting suitable music and designing tailored schedules for diverse patient populations. Therefore, while art therapy effectively serves as a non-pharmacological psychotherapeutic form, it lacks the capacity to treat numerous individuals simultaneously. We propose a potentially transformative conjecture: Combining Artificial Intelligence (AI) technologies could bring substantial impacts to the field of art therapy, enhancing its effectiveness, adaptability, and inclusiveness. However, given AI's nascent nature, extensive investigations and empirical analyses are necessary to fully explore and substantiate AI's potential application in art therapy.

The present review is subject to several limitations that warrant acknowledgment. Firstly, the investigation was restricted to studies written in English, thus limiting our sample range. Therefore, it is possible that we missed valuable insights from studies published in other languages. Consequently, our findings may not entirely reflect or apply to non-English speaking populations, introducing potential research bias.

Secondly, the term “mental disorders” encompasses a diverse range of diagnostic categories, implying the presence of unique differences within this group, which our reviewed studies might not have comprehensively explored. While our review attempted to provide a broad perspective, it is important to consider these inherent variations.

Thirdly, the rapidly evolving policies in different countries pose a formidable challenge to literature selection for reviews like this. To mitigate a scarcity of pertinent literature, we selected references applicable and discussable at the time of writing. Nevertheless, future studies may need to update the included literature, reflecting sudden changes in respective countries' policies.

Lastly, considering the small sample size and limited number of studies, we advocate for a prudent interpretation of the qualitative results. The conclusions drawn might not be fully representative of the broader population due to our sample's limited size. It is worth noting that only four studies were evaluated as excellent, further limiting the robustness of our conclusions. This limitation might potentially create a gap in our understanding of arts therapies' full potential in the global response to the COVID-19 pandemic.

## 5 Conclusions

In conclusion, based on the available literature, arts therapies have demonstrated long-standing effectiveness as a mental health intervention, suggesting potential advantages in addressing mental health concerns among individuals affected by COVID-19. Furthermore, scholarly investigations have indicated that it also plays a significant role in mitigating stress levels among healthcare professionals. The present study provides additional support for the efficacy of arts therapies in promoting the mental wellbeing of individuals affected by COVID-19, as evidenced by our analysis of Randomized Controlled Trials (RCTs). The results of our overlay visualization experiment reveal a notable shift in research emphasis from individual mental disorders to the wider mental health consequences of the COVID-19 pandemic. Additionally, there is a renewed scholarly interest in exploring the potential therapeutic benefits of meditation. The present analysis cautiously examines the potential efficacy of arts therapies as a treatment modality for mental health concerns arising from COVID-19. However, it is imperative to conduct additional specialized research to establish the validity of this assumption conclusively.

## Author contributions

XL: Conceptualization, Data curation, Formal analysis, Investigation, Methodology, Project administration, Resources, Software, Supervision, Validation, Visualization, Writing – original draft, Writing – review & editing. ZZ: Writing – original draft. XS: Writing – original draft. CH: Writing – review & editing. JW: Supervision, Writing – review & editing, Conceptualization, Data curation. QW: Investigation, Writing – review & editing, Supervision, Conceptualization, Data curation, Formal analysis, Funding acquisition, Methodology, Project administration, Resources, Software, Validation, Visualization. GH: Investigation, Writing – review & editing, Funding acquisition.
